# In Vivo Confocal Microscopy Evaluation in Patients with Keratoconus

**DOI:** 10.3390/jcm11020393

**Published:** 2022-01-13

**Authors:** Alvin Wei Jun Teo, Hassan Mansoor, Nigel Sim, Molly Tzu-Yu Lin, Yu-Chi Liu

**Affiliations:** 1 Department of Cornea and External Eye Disease, Singapore National Eye Centre, Singapore 168751, Singapore; alvin.teo@mohh.com.sg; 2 Al Shifa Trust Eye Hospital, Jhelum Road, Rawalpindi 46000, Pakistan; hassan-mansoor@hotmail.com; 3 Yong Loo Lin School of Medicine, National University of Singapore, Singapore 168751, Singapore; sim.nigel@u.nus.edu; 4 Tissue Engineering and Cell Therapy Group, Singapore Eye Research Institute, Singapore 169856, Singapore; molly.lin.t.y@seri.com.sg; 5 Cornea and Refractive Surgery Group, Singapore Eye Research Institute, Singapore 169856, Singapore; 6 Ophthalmology and Visual Sciences Academic Clinical Program, Duke-NUS Medical School, Singapore 169857, Singapore

**Keywords:** keratoconus, corneal nerves, in-vivo confocal microscopy (IVCM), cornea cross-linking (CXL), corneal sensitivity

## Abstract

Keratoconus is the most common primary corneal ectasia characterized by progressive focal thinning. Patients experience increased irregular astigmatism, decreased visual acuity and corneal sensitivity. Corneal collagen crosslinking (CXL), a minimally invasive procedure, is effective in halting disease progression. Historically, keratoconus research was confined to ex vivo settings. In vivo confocal microscopy (IVCM) has been used to examine the corneal microstructure clinically. In this review, we discuss keratoconus cellular changes evaluated by IVCM before and after CXL. Cellular changes before CXL include decreased keratocyte and nerve densities, disorganized subbasal nerves with thickening, increased nerve tortuosity and shortened nerve fibre length. Repopulation of keratocytes occurs up to 1 year post procedure. IVCM also correlates corneal nerve status to functional corneal sensitivity. Immediately after CXL, there is reduced nerve density and keratocyte absence due to mechanical removal of the epithelium and CXL effect. Nerve regeneration begins after 1 month, with nerve fibre densities recovering to pre-operative levels between 6 months to 1 year and remains stable up to 5 years. Nerves remain tortuous and nerve densities are reduced. Corneal sensitivity is reduced immediately postoperatively but recovers with nerve regeneration. Our article provides comprehensive review on the use of IVCM imaging in keratoconus patients.

## 1. Introduction

Keratoconus is an ectatic condition of the cornea that is characterised by progressive thinning and steepening, causing significant visual morbidity. Reported prevalence ranges from 0.3 to 3300 per 100,000, depending on diagnostic criteria and geographic location [1]. The pathophysiology of keratoconus is multifactorial. Environmental (microtrauma), genetics, and biochemical factors play a role in disease [1]. Eye rubbing is one of the important environmental factors of keratoconus. Repetitive, prolonged and greater force of eye rubbing is associated with its progression [2]. Patient factors include atopy such as asthma and hay fever [3], and usage of contact lens wear [4,5]. As for genetic factors, alterations in Lysyl oxidase (LOX), Collagen Type V Alpha 1 Chain (COL5A1), and Forkhead box protein O1 (FOXO1) gene have been correlated to keratoconus pathogenesis [6,7,8]. Other studies have also shown that relatives of patients with keratoconus have a high prevalence of undiagnosed keratoconus [9,10]. In addition, biochemical factors such as increased protease activity cause collagen cross-linkages in the stroma to be broken down [11].

There has been much interest in corneal nerve structure, function and their role in corneal health and disease [12]. Corneal nerves beside their sensory function also secrete neuromediators that are vital to the development and maintenance of the cornea. It is hence important to understand the function and morphology of corneal nerves in diseased states. In keratoconus, attempts to understand corneal nerves were previously confined to ex vivo studies or cornea buttons with severe disease with staining techniques [13]. Most recently, the use of confocal microscopy in analysing keratoconic corneas have been instrumental in understanding the microstructural changes in vivo.

In vivo confocal microscopy (IVCM) is a non-invasive imaging modality that has been used to examine and quantify the cellular structure of the cornea in vivo [14,15]. It attains images by optical sectioning, where a light is focused via a small aperture onto the tissue, and in focus light is processed while light from out of focus planes are attenuated. The term “confocal” means that there is a common focal point between the illumination and collection systems. An en face image can be processed once the scan proceeds serially through the cornea depth. This allows microstructures such as corneal epithelium, and stromal keratocytes to be imaged at a cellular level [16,17]. Although the field of view of a single image is small (typically 0.16 mm^2^), multiple IVCM images can be constructed into a mosaic image using automatic tissue classification algorithms for large-area visualisation and analysis [18]. The laser scanning confocal microscope is the most advanced of these and is the only design that is commercially available currently. It achieves 800 times magnification, lateral resolution of 1 µm, and axial resolution of 4 µm [14]. IVCM has thus emerged as a promising tool to study ocular and systemic diseases causing corneal neuropathies [19]. With the advancement of analytic tools, it allows for reliable longitudinal assessment on corneal nerve changes with good measurement repeatability and reproducibility [20,21,22].

The introduction of crosslinking in 2003 provided a minimally invasive treatment option for patients with keratoconus to halt disease progression [23]. This procedure has also shown good long-term results, effectively halting the progression of corneal ectasia, with stabilization of refractive status and topographical changes [24,25,26]. In conventional protocols, the epithelium is removed for better riboflavin and UV-A absorption. Other variations such as transepithelial CXL in which the corneal epithelium is left intact, have also been suggested to reduce the risk of infection, improve postoperative patient comfort and aid visual recovery.

Many studies have now depicted corneal nerve changes in the keratoconic cornea before and after crosslinking using in vivo confocal microscopy images. With increasing recognition of the important role corneal nerves play in maintaining structure and function of the cornea, we aim to summarize the literature regarding the use of in vivo confocal microscopy in the keratoconic cornea before and after CXL in this review. Aspects related to corneal nerve morphology, corneal sensation, and protocols in CXL are presented.

## 2. Systematic Review Methodology

Four international databases (Web of Science, PubMed, Scopus, and Google Scholar) were searched for relevant articles. All cross-sectional and longitudinal studies discussing keratoconus, cross linking and corneal sensitivity in the body, figures, or tables of the article were accepted without any restrictions.

### 2.1. Search Strategy

Key words such as “keratoconus”, “corneal sensitivity”, “cross-sectional studies”, “longitudinal studies”, “in vivo confocal microscopy” “cornea collagen cross-linking”, and “corneal nerves” were used to search the databases of Web of Science, PubMed, Google Scholar, and Scopus from inception to December 2021. Relevant articles had their reference lists reviewed for articles of interest as well.

### 2.2. Inclusion Criteria

All stages of the study followed the PRISMA guidelines. Observational epidemiological studies including cross-sectional, case–control, and cohort studies that had a population-based design were included in the study. If several studies were conducted in a certain population, the higher quality study was included in the analysis. Studies from 2010 were preferably chosen to ensure the review is updated. Studies which did not meet one or more inclusion criteria were excluded from the study. The outcome of the study was the function and morphology of corneal nerves, in vivo confocal microscopy, collagen crosslinking and corneal sensitivity.

Two reviewers (A.W.J.T. and Y.C.L.) screened all retrieved articles by title and abstract initially. Only original research articles written in English were included. Analysis reviews, editorials, opinions were excluded. The articles retrieved were then curated manually to assess relevance to the study’s objective. Additionally, the reference lists of remaining studies were checked to identify further relevant articles that may have been overlooked during the initial process. All the eligible articles were obtained and fully read.

We excluded articles where IVCM findings were not mentioned in the results of the full text article. Studies where recovery of full text was not possible, even after searching the available medical databases and/or contacting the corresponding authors, were excluded. Disagreements were settled through discussion with an expert for arbitration.

### 2.3. Data Extraction and Quality Evaluation of the Studies

The initial database search with the above keywords identified 265 papers. After excluding articles where full text was not available (21), 244 articles were left. After going through title and screening through the abstract and applying our inclusion/exclusion criteria (26 were reviews) 218 studies were left. After full text-retrieval and further curation, 84 studies remained.

## 3. Corneal Nerve Function and Anatomy

**Figure 1:**
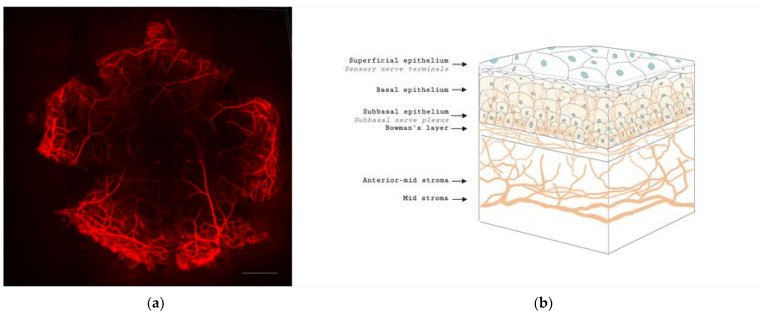
Anatomy of corneal nerves. (**a**) Whole mount staining with anti-class β III tubulin of mice cornea showing the distributions of corneal nerve. Scale bar: 500 μm. (**b**) Cross section of corneal nerves. (**b**) is created by Biorender.

The cornea is a highly innervated structure. Corneal nerves originate from the ophthalmic branch of the trigeminal nerve [27]. The main stromal nerve bundles enter the human cornea radially at the corneoscleral limbus at a distance of 293 ± 106 µm from the ocular surface and are distributed uniformly throughout the corneal circumference [28]. Soon after entering the cornea, each stromal nerve bundle gives rise through repetitive branching to varying numbers of progressively smaller and smaller stromal nerves that anastomose frequently, often at highly acute branch points, to form a moderately dense midstromal plexus. Most midstromal nerve fibres turn abruptly 90 degrees and continued into the narrow band of anterior stroma located immediately beneath bowman’s membrane, and gives rise to a dense, roughly two-dimensional, subepithelial plexus [29]. The subepithelial plexus has a characteristic plexiform appearance due to the anastomosis of tortuous nerve fibres, with it being denser in the peripheral and intermediate cornea than the central region. Straight fibres from the subepithelial plexus generally penetrate Bowman’s membrane and continued into the corneal epithelium, with other nerves becoming subbasal nerves that course parallel to the ocular surface near the interface of Bowman’s membrane and the basal epithelium (Figure 1). Subbasal nerves form a gentle spiral-like clockwise assemblage of long, curvilinear nerve fibres that converge on an imaginary center, or vortex, located inferior and slightly nasal to the corneal apex. This assembly is believed to be influenced by the electromagnetic fields of the eye [30]. They then form intraepithelial terminals that are distributed abundantly throughout the epithelium.

Corneal nerves have afferent and efferent function, conveying touch and pain, as well as producing neuromediators such as neurotrophins and neuropeptides that is thought to play a role in its pathophysiology. These serve as trophic factors in ocular homeostasis and maintaining corneal microstructure. Corneal epithelial, stromal cells and endothelial cells also contribute to the diversity of neuromediators in the cornea by producing neurotrophins [31]. Neurotrophins, such as nerve growth factor (NGF), regulate neuronal development, survival, death and plasticity [12]. In keratoconus, the high affinity receptor of NGF, tyrosine kinase receptor A, was found in high levels and is thought to be due to heterologous upregulation for maintenance of unmyelinated corneal nerves [32]. Another neurotrophin, ciliary neurotrophic factor (CNTF) which is important for protection of the cornea from oxidative radical damage, had a higher expression of its mRNA in keratoconus as compared to normal eyes [32].

Neuropeptides are released slowly, act over an extended period, involved in neurotransmission and have a paracrine function. Calcitonin gene-related peptide (CGRP) plays an important role in the nociceptive pathway in the cornea, by activating factors such as bradykinin and stimulating the release of nitrous oxide [33]. These effects help produce a favorable neurochemical environment that enhances neural activity. Vasoactive intestinal peptide (VIP) is another important neuropeptide, playing a role in corneal wound healing [34] by exerting anti-inflammatory effects in a signaling pathway dependent manner [12,35]. Work by Sacchetti and colleagues analysed 12 keratoconic corneas obtained post keratoplasty and found that keratoconic corneas showed significantly higher CGRP and VIP levels as compared to controls. This increase is thought to be due to an attempt by sensory nerves to counteract degenerative changes in keratoconus [36].

## 4. Cellular and Corneal Nerve Morphological Changes in Keratoconus

### 4.1. Microstructural Changes

A lack of animal models for keratoconus renders investigation into the cellular changes difficult, and excised corneas usually represent severe disease. In ex-vivo studies, Brookes et al., found an increase in enzymatic activity in stromal keratocytes with immunohistochemical staining, and this change leads to destruction of the cornea [13]. In a study analysing corneal buttons with severe keratoconus using the acetylcholinesterase technique, stromal nerves were thickened, increasingly tortuous and disorganised with looping and coiling. Subbasal nerves showed loss of their radial, clockwise whorl configuration with tortuosity and localized thickening [37]. These staining techniques, however, can only be applied to in-vitro or ex-vivo corneas but not study corneas in vivo [38].

**Figure 2:**
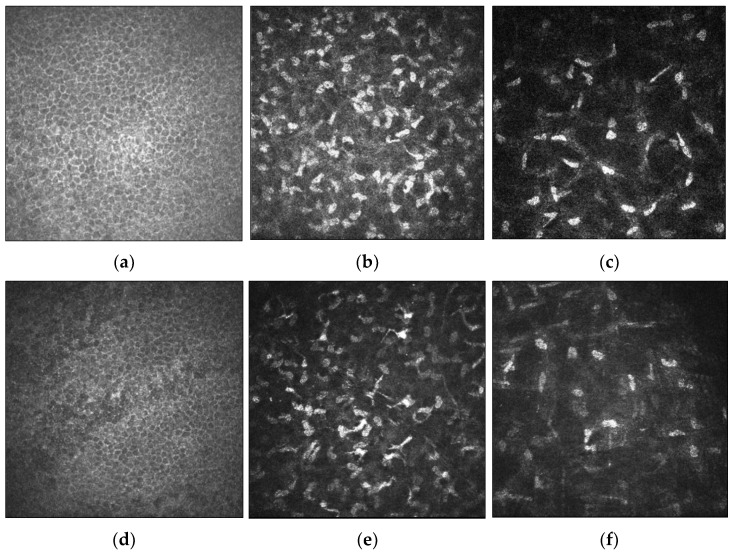
In-vivo confocal microscopy (IVCM) imaging of the corneal epithelium, anterior stromal keratocytes and posterior stromal keratocytes in healthy (**a**–**c**, respectively) and keratoconic eyes (**d**–**f**, respectively). Cell densities of the corneal epithelium, anterior stromal keratocytes and posterior stromal keratocytes are reduced in keratoconic eyes relative to healthy subjects. Scale bar: 100 µm.

Several changes of the corneal cell and nerve microstructure in patients with keratoconus have been observed on IVCM images (Figure 2). Corneal stromal keratocytes (CSKs) are a population of quiescent mesenchymal-derived cells residing between collagen lamellae [39]. The cell density is the highest in the anterior 10% of the stroma and decreases posteriorly. CSKs possess dendritic processes to connect with neighboring cells, forming a highly organized syncytium throughout the stroma [40]. Some keratocytes are located in the vicinity of stromal nerves and occasionally enwrap nerve fibres with cytoplasmic extensions, suggesting an interdependence of the two [29]. Studies comparing keratoconic corneas and healthy controls found that there was generally lower stromal keratocyte density (Figure 2e,f) [41,42], with pronounced reflectivity and irregular arrangement of the stromal keratocytes [41,43]. There is loss of corneal stromal thickness over time, postulated to be due to a release of degradative enzymes [44]. These changes in cell densities may also be secondary to other factors such as contact lens wear [42,45,46].

**Figure 3:**
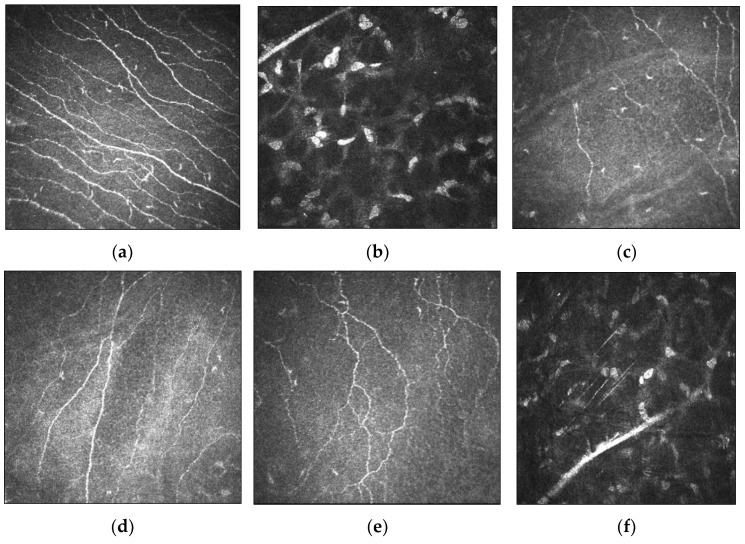
Morphology of corneal nerves evaluated by IVCM imaging in healthy and keratoconic corneas. (**a**) Subbasal nerve plexus with almost parallel nerve fibre bundles as observed in healthy corneas. (**b**) Normal stromal nerves in healthy corneas. (**c**–**e**) IVCM images demonstrate decreased nerve fibre density, thickened subbasal nerves and tortuous nerve paths in keratoconic corneas, respectively. (**f**) Thickened stromal nerves in keratoconic corneas. Scale bar: 100 µm.

IVCM images of the corneal nerve plexus in keratoconic eyes showed that nerve fibre bundles were tortuous and formed closed loops within the apex of the cone. In severe keratoconus, there was abrupt termination of the nerve fibres in the region of the cone. The subbasal nerve architecture was also abnormal, with predominantly oblique and horizontal orientation of subbasal nerve fibres at the apex, and the curvilinear orientation at the base of the cone differed markedly from the normal inferocentral whorl-like region seen in the normal cornea. Mean densities were also reduced at 10,478 ± 2188 µm/mm^2^ as compared to normal corneas (21,668 ± 1411 µm/mm^2^) [47]. An increase in nerve fibre tortuosity and diameter were observed [42,46]. The mean diameter of stromal nerve fibres was reported to be significantly greater in subjects with keratoconus compared to control subjects (10.2 ± 4.6 µm versus 5.5 ± 1.9 µm) [46]. These findings were supported in a larger study of 145 patients in which participants were stratified into the manifest keratoconus group, the subclinical keratoconus group, the relatives of keratoconus group and the control group. They found that there was no significant difference between the subbasal nerve diameter amongst all groups, but the mean stromal nerve diameter in all three keratoconus groups (8.0 ± 3.5 µm to 8.8 ± 3.5 µm) was significantly higher than the control group (5.4 ± 2.1 µm; *p* < 0.001) [48]. Enlargement of nerves were thought to be related to impairment of nerve function, while increased nerve tortuosity was a morphologic marker of nerve regeneration [49]. A comparison of corneal nerve morphology between healthy and keratoconic corneas is demonstrated in Figure 3.

Changes to the corneal nerve fibre length and subbasal nerve plexus were also noted in patients with keratoconus. The nerve fibre length was reduced significantly (16.4 ± 1.9 mm/mm^2^) compared to healthy corneas (23.8 ± 3.3 mm/mm^2^), and the subbasal nerve plexus was significantly more tortuous [50].

There is an increased risk in the first 6 years for young, unilateral keratoconus patients with a normal eye developing keratoconus in that eye subsequently [51,52]. However, our current imaging tools have not yielded any methods used to screen for keratoconus efficiently. IVCM analysis so far does not show any predictive factors. Studies analysing the fellow normal eye of a patient with keratoconus in one eye compared with normal controls showed that there were no significant differences between corneal nerve fibre densities, length, and branch densities [53]. Another study showed that there was a significant difference in the stromal keratocyte densities of subclinical keratoconus and controls, but there were no significant differences in subbasal nerve densities and diameters [48].

The corneal cellular and nerve changes in keratoconus are summarized in Table 1.

### 4.2. Relationship between Corneal Nerves and Corneal Sensitivity in Keratoconus

Evaluation of corneal sensitivity in diseased states is important as it serves as a functional measure of corneal nerves, which have important role in maintaining normal cellular structure and function as described earlier. However, it is known that clinical function and nerve alterations may not always correlate. Clinical symptoms can be present in the absence of visible nerve pathology and vice-versa [12].

Despite substantial nerve remodeling, the effect on corneal sensitivity is equivocal. Early studies using the Cochet–Bonnet aesthesiometer suggested that corneal sensitivity in keratoconus patients decreased in proportion to worsening disease severity [56,57]. However, the contact aesthesiometer is relatively crude and has certain limitations such as a limited stimulus range and an inability to distinguish subtle changes in corneal sensitivity [58,59]. A newer way of measuring corneal sensation such as the Belmonte non-contact gas aesthesiometer was developed to overcome these limitations. Using gas aesthesiometry, corneal sensation was found to be significantly reduced in keratoconus patients with mechanical, chemical and thermal stimulation, independent of severity of disease [60]. Hence, results in the literature may vary with the use of different aesthesiometers. Moreover, the use of rigid contact lens, a common management for keratoconus, is a confounding factor. The contact lens is known to reduce corneal sensitivity in normal and keratoconus corneas [61,62]. This has given rise to varying results, with some studies showing reduced corneal sensation in keratoconus patients [57,60,62], while some showed no difference between keratoconus patients and controls after adjusting for contact lens wear [54,55].

## 5. Corneal Nerve and Cellular Changes, and Corneal Sensitivity after Crosslinking

### 5.1. Corneal Nerve and Cellular Changes

A number of studies showed initial nerve degeneration that occurred immediately after CXL but nerves regenerated over time [63,64,65]. In a rabbit study, Xia et al., showed an initial absence of subepithelial nerve plexus with nerve fibre debris and nerve degeneration within 7 days of undergoing epithelium-off CXL. Fine nerve fibres were then found to be sprouting from neighboring non-injured nerve fibres in the deeper corneal stroma 7 days after CXL. The regenerating nerves made a tortuous progression toward the centre of the cornea to penetrate the denervated areas. They were found in excess throughout the anterior stroma, with the corneal nerve fibre density returning to normal levels by 180 days. Although there was a reduction in corneal sensitivity in the first 7 days after CXL, significant corneal nerve regeneration resulted in restoration of corneal sensitivity 90 days after the procedure. Rabbit corneas that underwent transepithelial CXL had no changes to the corneal nerves [63].

**Figure 4:**
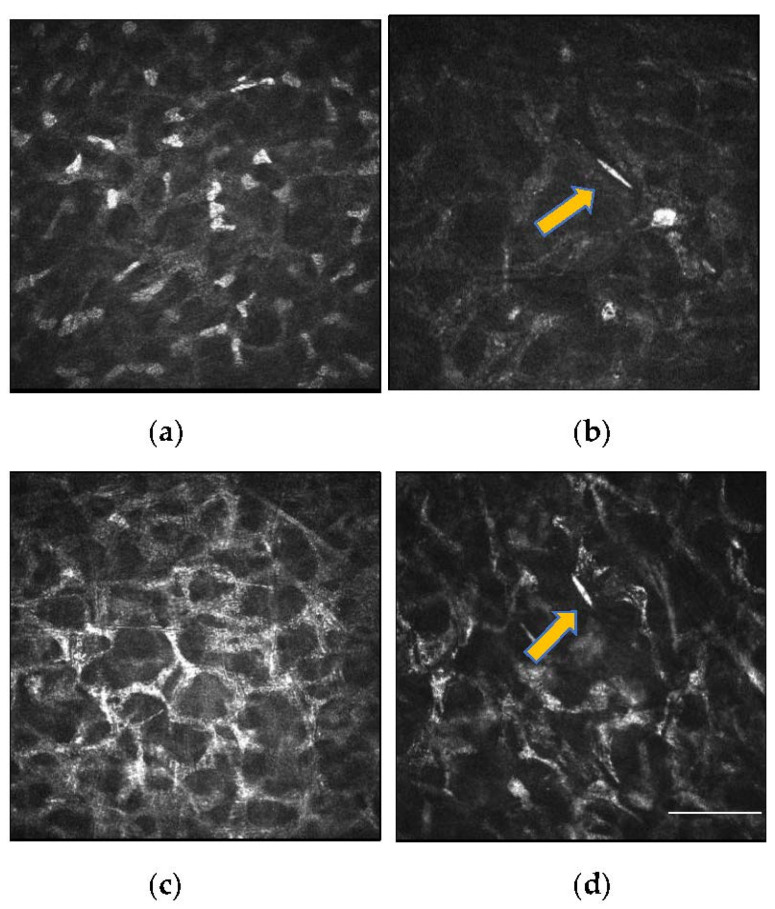
IVCM images of the anterior corneal stroma after CXL. (**a**) Rarefaction of keratocytes and elongated nuclei (masked necrotic keratocytes) are observed. (**b**) Reduction in keratocyte density with the presence of a fine needle-like opacity (yellow arrow), suggestive of apoptosis of keratocytes. (**c**) Anterior stromal honeycomb similar to edema, comprising of hyper-reflective cytoplasm and extracellular lacunae are evident (**d**). Repopulation of the cross-linked area with activated keratocytes. A needle-like opacity (yellow arrow) is also detectable, indicating apoptotic keratocytes. Scale bar: 100 µm.

**Figure 5:**
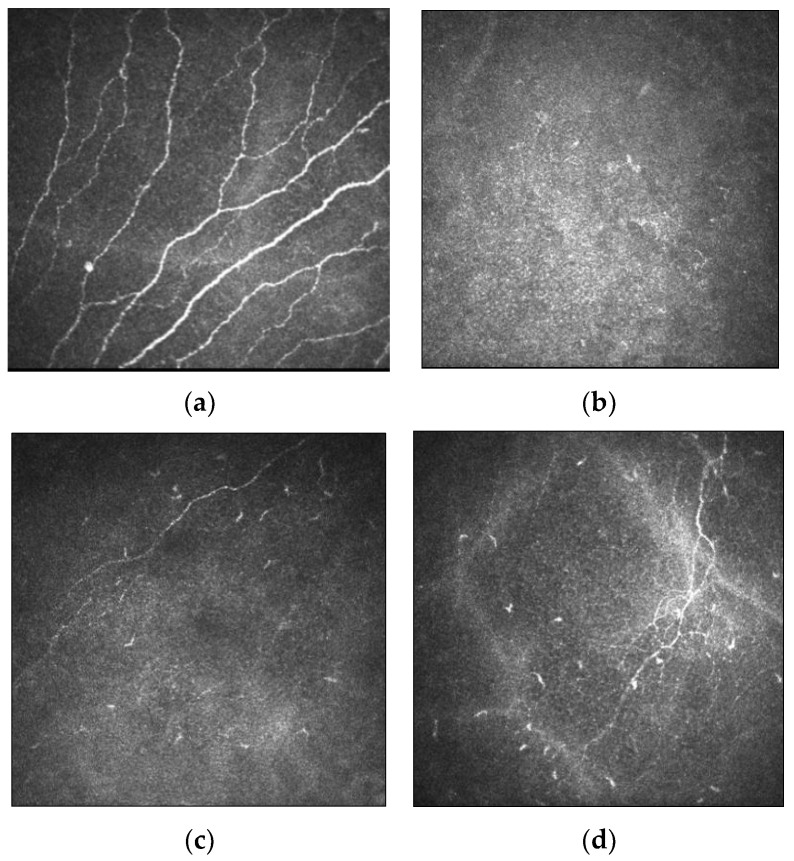
IVCM images demonstrating corneal nerve architecture before and after CXL. (**a**) Thickened subbasal nerves as noticed in a keratoconic cornea before CXL. (**b**) There is decreased nerve fibre density 1-month post-CXL. (**c**) At 4-months after CXL, there is an initial re-innervation process characterized by sprouting nerve fibres. Langerhans cells are also detectable, suggestive of transient post-CXL inflammation. (**d**) Increased nerve fibre density and tortuosity 1-year after CXL. Scale bar: 100 µm.

Clinical studies subsequently analysed IVCM images of keratoconus patients who underwent CXL at intervals of 1, 3, 6-months and 1-year post procedure. At one month after CXL, there is rarefaction of keratocytes associated with honeycomb-like stromal edema in the anterior 300 μm of the cornea (Figure 4). Hyper-reflective microparticles, representing keratocyte apoptotic bodies, are also visible (Figure 4d) [64]. After 3 months, there is keratocyte repopulation with increased density of the extracellular matrix and resolution in stromal haze after 3 months. There is also collagen compaction by new structured fibres in the anterior-mid stroma [64,65,66]. The subepithelial nerve fibres regenerated more rapidly than the stromal nerve fibres from the surrounding non-irradiated area between the second and third postoperative months. At 6 months postoperatively, there is a dense keratocyte population with increased extracellular matrix density. Subepithelial nerve regeneration is almost complete with restored corneal sensitivity [65,67]. However, not all eyes follow the same timeframe, with a study finding a slight delay in the regeneration of the subepithelial plexus in 68.8% of eyes at 6 months after CXL [68]. In another study, disconnected neural fibres were observed under the Bowman’s lamina 6 months post-CXL [64]. However, the number of fibres increased progressively, and interconnections began to resemble the preoperative sub-epithelial plexus structure 12 months after CXL. The nerve fibre regeneration process is characterized by the presence of native subepithelial nerve flocks simulating Langerhans cells in a “pseudodendritic pattern”. Langerhans cells were detectable between the second and third month after CXL, suggesting transient postoperative inflammation or an initial reinnervation process characterized by sprouting nerve fibres (Figure 5) [64]. At 12 months, the subepithelial nerve plexus and densities recover to preoperative values with repopulation of keratocytes [69,70].

In a 5-year prospective study, patients with early-stage keratoconus who underwent conventional CXL had similar changes in the first year as described earlier. After 1 year, there was a continual increase in the median nerve fibre density with nerves adopting configurations of increasing loops, crossings and tortuosity. They also adopted radial, circumferential, or mixed orientations as they regenerated. Final nerve densities matched preoperative nerve densities but remained reduced relative to healthy corneas [71] (Figure 5).

In corneas thinner than 400 μm, it was traditionally thought to be a contraindication due to the potential for endothelium toxicity. Various methods utilizing contact lens [72], Hypotonic riboflavin solution [73,74], and epithelial island cross linking techniques [75] have been used to overcome this limitation. The effect on corneal nerves were found to be similar as compared to conventional CXL protocols. There was an absence of the subbasal nerve plexus and significant keratocyte apoptosis in the first postoperative month. By six months, near total recovery of the subepithelial nerve plexus had occurred [72,73]. Anterior stromal keratocyte density were reduced with corneas showing significant keratocyte apoptosis [73,75]. There was gradual recovery of keratocytes but this did not reach pre op levels (572 vs. 368, *p* < 0.007) at the end of 6 months [73]. Endothelial cell density were similar pre- and post-operatively in contact lens assisted CXL and epithelial island CXL [72,75] but there was a decrease in protocol utilizing hypotonic riboflavin from 2895 to 2660 (*p* < 0.005) [73]. Endothelial cell morphology remained the same, with no corneal edema [73]. While these results are promising, they are limited by their small study population and relatively short study follow up, with long term studies needed to prove their safety and efficacy.

The effect on corneal nerves following accelerated CXL or transepithelial CXL has also been studied. The subbasal nerve densities of 153 eyes undergoing accelerated and conventional epithelium-off CXL were investigated using IVCM images. There was a significant decrease in subbasal nerve density of the conventional CXL group than the accelerated CXL throughout the study period except on the final visit of 15 months postoperatively. This difference was thought to be due to the longer time of ultraviolet light exposure in the conventional protocol [76].

Studies evaluating the effect of transepithelial CXL on corneal nerves show less consistent results. Studies found that unlike the conventional epithelium-off treatment, subepithelial and anterior midstromal nerve fibres in transepithelial CXL remained present [77,78]. On follow-up visits within 6 months with IVCM, the nerves showed increased reflectivity with a granular appearance, and had irregular paths with branch anomalies. However, other studies report a significant decrease in the number of nerve fibres at one month after transepithelial CXL, with recovery to pre operative densities at 6 months [79,80,81]. This suggests that mechanical removal of the epithelium in CXL is not the only explanation for the reduction in corneal nerve densities. CXL itself may have a role in altering the cornea nerve plexus. Subsequent studies used iontophoresis, a technique used to drive negatively charged riboflavin across the intact epithelium, in transepithelial crosslinking protocols [79,82]. With regards to the corneal stroma, lacunar edema, apoptotic keratocytes and activated keratocytes with elongated membrane processes are seen 3 months postoperatively. The effect of newer variations on corneal nerves such as pulsed transepithelial CXL [83] or the usage of supplemental oxygen is yet to be investigated [84]. Table 2 summarises the main studies investigating corneal nerve and cellular changes after CXL in keratoconus.

### 5.2. Changes in Corneal Sensitivity in Relation to Corneal Nerve Status after CXL

Besides evaluating corneal nerve metrics, ocular surface sensitivity and integrity are functional measures of corneal nerve status. Wasilewski and colleagues analysed corneal tactile sensitivity using the Cochet–Bonnet aesthesiometer in patients after CXL. The median sensitivity was 53.0 ± 8.7 mm preoperatively, 20.0 ± 16.2 mm at 7 days, 33.0 ± 16.4 mm at 30 days, 40 ± 12.6 mm at 90 days and 45 ± 9.2 mm at 180 days [87]. Decreased sensation was thought to be due to removal of the epithelium, and recovery of sensation was thought to correlate to nerve regeneration as described earlier in this review. In another study reporting the time course of ocular surface sensitivity changes using the Cochet–Bonnet aesthesiometer, the mean sensitivity was 59.0 ± 3.0 mm before CXL, decreased to 52.0 ± 13.0 mm at 3 months, and recovered to preoperative levels at 6 months with no further change at 12 months and at 5 years [71].

With regards to accelerated CXL, a study showed that the mean corneal sensation, measured by the Cochet–Bonnet aesthesiometer, decreased from 56.0 ± 5.4 mm before surgery, to 11.0 ± 4.5 mm and 33.0 ± 10.3 mm, in the first and third month after CXL, but recovered to preoperative values at 6 months. The mean subbasal nerve densities were significantly decreased up to 6 months postoperatively and recovered to preoperative levels only 12 months after procedure. This suggested that the recovery of corneal sensitivity preceded recovery of subbasal nerve densities to preoperative levels [85], implying that clinical function and nerve morphology may not always correlate.

Tolerance to RGP lenses after CXL has also been investigated by comparing corneal sensation, corneal nerve changes and lens wearing times. The mean corneal sensation, assessed by the Cochet–Bonnet aesthesiometer, decreased from 0.44 ± 0.05 g/mm^2^ to 1.19 ± 0.72 g/mm^2^ at 1 month, but improved to 0.48 ± 0.06 g/mm^2^ and 0.44 ± 0.05 g/mm^2^ at 3 to 6 months postoperatively. No subepithelial plexus could be visualised at one month but there was gradual restoration of corneal innervation with comparable preoperative levels at 6 months. Patients were more tolerant of RGP lenses with increased wearing times at the end of the 6-month study. Contribution of the flattening effect of CXL and a potential decrease in corneal sensitivity was thought to improve wearing of contact lenses [86].

## 6. Future Applications of IVCM in Keratoconus

IVCM images have been thought to be usable as a screening tool in patients with diabetic corneal neuropathy. Corneal nerve length and thickness have been reported to be early markers of eye involvement in patients with type 2 diabetes [88]. With the incorporation of deep learning techniques, artificial intelligence-based algorithm could provide rapid and good localisation performance for the quantification of corneal nerve biomarkers [89]. At this time of writing, there has not been any articles utilizing artificial intelligence techniques to analyse IVCM images in keratoconus. Although the prevalence of keratoconus is less than diabetes, we believe it could play a supplemental role to the armament of methods used to screen keratoconus.

The evaluation of subclinical or forme fruste keratoconus currently does not have any consensus. Although advances in corneal tomography and biomechanical assessments have made keratoconus diagnosis easier in the early stages, evaluation of these cases remain challenging [90]. Current evidence in the literature using IVCM images of corneal nerves taken from eyes with forme fruste keratoconus is limited. Larger study populations with well-defined inclusion criteria would possibly allow us to better understand nerve changes occurring in this subset of patients with very early keratoconus and possibly provide an opportunity for screening.

As described earlier, neuromediators secreted by corneal nerves play an important role in corneal health. There have been attempts to correlate neuromediator profiles with the severity of keratoconus [91]. We postulate that analysis of IVCM images along with neuromediator profiles and proteomic or metabolomic studies may uncover new insights into the pathophysiology of keratoconus.

## 7. Conclusions

Keratoconus presents with cornea ectasia that causes significant visual disability from a young age. Recent research has shown the possible role of corneal nerves in the pathophysiology of the disease. Aside from clinical examination, keratometric, topographical and biomechanical assessments that demonstrate clinical severity, IVCM has allowed accurate and reliable in-vivo evaluation of keratoconus at a cellular level, replacing the need for pathologic studies to understand the cellular and tissue changes. On IVCM evaluation, keratoconic corneas showed lower stromal keratocyte densities, thicker corneal nerves, reduced nerve fibre length, increased nerve tortuosity and irregular orientation, leading to decreased corneal sensitivity. However, the decreased sensitivity may not be positively correlated with the severity of the disease. Immediately after CXL, the subbasal nerve plexus, anterior and mid-stromal nerve densities were significantly reduced in the first six months, but these recovered gradually with restoration to preoperative levels by 12 months. The continued study of keratoconus with IVCM will allow us to further investigate the role that corneal nerves play in its pathophysiology, as well as the corneal nerve changes secondary to keratoconus. This has potential to allow further treatments on modulating corneal neuropathic changes to be developed in the future.

## Figures and Tables

**Table 1:** Results of main studies investigating cellular and corneal nerve changes in keratoconus.

Author	Assessment	Number of Eyes	Findings
Brookes et al. [13] 2003	Excised corneas	10 KCN, 3 controls	Using immunohistochemistry, localised nerve thickenings and anterior keratocyte nuclei were seen wrapping around corneal nerves—postulated to play a role in disease pathology.
Aqaba et al. [37] 2011	Excised corneas	14 KCN, 6 controls	Using acetylcholinesterase staining technique, 71% of keratoconic corneas demonstrated central stromal nerve changes such as thickening, tortuosity, nerve spouting and overgrowth.
Mocan et al. [42] 2008	IVCM assessment	68 KCN, 22 controls	Lower anterior stromal, mid-stromal and posterior stromal keratocyte density, lower endothelial cell density, subbasal long nerve density and thicker corneal nerves were found in keratoconus.
Patel et al. [47] 2006	IVCM assessment	4 KCN	Abnormal subbasal nerves with a tortuous network of nerve fibre bundles were present at the apex. Central subbasal nerve density was significantly lower in keratoconus corneas.
Flockerzi et al. [50] 2020	IVCM assessment	23 KCN	Subbasal nerves are shorter and are more tortuous in the keratoconus cornea.
Mannion et al. [46] 2007	IVCM assessment	1 KCN	Thicker nerve fibre bundles in the stroma and reduced nerve fibre density were found in the subepithelial plexus of the keratoconus cornea.
Mannion et al. [54] 2005	IVCM assessment	13 KCN, 13 controls	Mean diameter of nerve fibres in stroma was found to be greater in subjects with keratoconus compared to controls. There was altered orientation of the nerve fibres in keratoconus.
Ozgurhan et al. [48] 2013	IVCM assessment	30 KCN, 32 subclinical KCN, 53 KCN relatives, 30 controls	Stromal keratocyte densities were significantly lower in all KCN groups as compared to controls. Significantly higher mean stromal nerve diameter was noted in all KCN groups as compared to controls.
Patel et al. [55] 2009	IVCM assessment	27 KCN, 31 controls	Subbasal nerve density and basal epithelial density were significantly lower than controls in all keratoconic eyes.
Pahuja et al. [53] 2016	IVCM assessment	33 normal eyes of KCN, 30 controls	Significant difference in corneal nerve fibre densities and length between keratoconus eyes and control eyes. No significant difference between unaffected eye of keratoconus patient and controls

KCN, keratoconus; IVCM, in-vivo confocal microscopy.

**Table 2:** Results of main studies investigating corneal cell and nerve alternations after CXL in keratoconus.

Author	Study and CXL Protocol	N. of Eyes	Follow Up	Findings
Xia et al. [63] 2011	Longitudinal study, transepithelial or epithelium-off conventional CXL	108 rabbit eyes	180 days	Immediate reduction of corneal sensitivity and decrease in nerve density after conventional CXL. Gradual recovery to normal levels occurred at 90 days and 180 days respectively. Rabbits that underwent transepithelial CXL showed no significant difference in cornea sensitivity.
Mazzotta et al. [64] 2015	Longitudinal study; epithelium-off CXL	84 eyes	12 months	Regeneration of subepithelial and stromal nerves was complete with fully restored corneal sensitivity 12 months after CXL.
Mazzotta et al. [65] 2008	Longitudinal study; epithelium-off CXL	44 eyes	3 years	Immediate disappearance of subepithelial plexus and anterior-mid stromal nerve fibres after CXL, with restoration of nerve plexus and full corneal sensitivity at one year after CXL.
Parissi et al. [71] 2016	Longitudinal study; epithelium-off CXL	19 eyes	5 years	Nerves continued to regenerate 5 years after CXL but remained reduced relative to normal corneas. More nerve loops, crossings and greater crossing angles were observed.
Al-aqaba et al. [78] 2012	Cross-sectional study; transepithelial or epithelium-off CXL	8 eyes	N/A	Absence of subbasal nerves in the epithelium-off CXL group was attributed to mechanical removal of epithelium. Subbasal nerves were detected immediately after transepithelial CXL. Stromal nerves had localised swellings with disruption of axonal membrane and loss of axonal continuity within the treatment zone.
Zare et al. [68] 2016	Longitudinal study; epithelium-off CXL	32 eyes	6 months	At 1 month, subepithelial nerve plexus was absent in 25 eyes (78.1%) and was reduced in 7 eyes (21.9%). The plexus was absent in 22 eyes (68.8%) and reduced in 10 eyes (31.3%) at 6 months.
Jordan et al. [70] 2014	Longitudinal study; epithelium-off CXL	38 eyes	12 months	Mean subbasal nerve density decreased significantly at 1, 3, and 6 months, with a return to preoperative values at 12 months postoperatively.
Mazzotta et al. [72] 2016	Longitudinal study; contact lens assisted epithelium-off CXL	10 eyes	6 months	Corneal reinnervation was fully restored at 6 months. Keratocyte apoptosis occurred after the procedure but this recovered at 6 months. No changes to endothelial cell count.
Sufi et al. [73] 2021	Longitudinal study; epithelium-off CXL with hypotonic riboflavin	10 eyes	6 months	Absence of the subbasal nerve plexus at the first postoperative month. There was nearly total regeneration of subepithelial nerve plexus at end of 6 months. Anterior stromal keratocyte densities were reduced even at the end of 6 months. Endothelial cell densities decreased from 2895 to 2660 cells/mm^2^.
Mazzotta et al. [75] 2014	Longitudinal study; epithelial island CXL	10 eyes	12 months	Keratocyte apoptosis and nerve fibre loss under the epithelial island and de-epithelialized ring at 1 month postoperatively. No change in endothelial cell densities after the procedure.
Kymionis et al. [69] 2009	Longitudinal study; epithelium-off CXL	5 eyes	12 months	The subepithelial nerve plexus was absent within the CXL treatment zone at the first postoperative month. There was reinnervation at 3 months, with keratocyte repopulation at 6 months.
Hashemian et al. [76] 2014	Longitudinal study; epithelium-off or AXL	153 eyes	15 months	Anterior stromal keratocyte density and subbasal nerve density decreased significantly in AXL and CXL groups 1 month postoperatively. Both nerve parameters were significantly decreased in the conventional CXL group for 1 year but were comparable with AXL at 15 months.
Caporossi et al. [77] 2012	Longitudinal study; transepithelial CXL	10 eyes	6 months	Subepithelial and stromal nerve fibres were present immediately post procedure. There was limited apoptosis of keratocytes.
Bouheraoua et al. [81] 2014	Longitudinal study; transepithelial CXL, epithelium-off CXL or AXL	45 eyes	6 months	Compared to preoperative values, the mean corneal subbasal nerve and anterior stromal keratocyte densities were significantly lower at 6 months in the epithelium-off CXL and AXL groups. Postoperative values of subbasal nerve and anterior stromal keratocyte densities were comparable to the preoperative values in the transepithelial group.
Filippello et al. [80] 2012	Longitudinal study; transepithelial CXL	20 eyes	18 months	Stromal Keratocytes and nerve fibres decreased in number (approximately 25%) after transepithelial CXL. They returned to pretreatment levels about 6 months after the procedure.
Jouve et al. [79]. 2017	Longitudinal study; transepithelial CXL using iontophoresis or Epithelium-off CXL	80 eyes	24 months	Mean corneal subbasal nerve and anterior stromal keratocyte densities were significantly lower than preoperative values in both groups, but there was faster recovery to preoperative levels in the transepithelial group (6 months vs. 12 months).
Ozgurhan et al. [85] 2015	Longitudinal study epithelium-off AXL	30 eyes	12 months	Corneal sensitivity significantly decreased at 3 months but increased to preoperative ranges after 6 months. There was still a significant decrease in mean subbasal nerve fibre density at 6 months postoperative but restored to preoperative values at 12 months.
Unlu et al. [86] 2017	Longitudinal study; epithelium-off CXL	30 eyes	6 months	Mean corneal sensation decreased in the first month and recovered to preoperative levels at 6 months. Subbasal nerve plexus gradually regenerated to almost preoperative levels at 6 months.

CXL, corneal crosslinking; AXL, accelerated crosslinking.

## Data Availability

Not applicable.
